# Bovine tuberculosis in African buffaloes: observations regarding *Mycobacterium bovis *shedding into water and exposure to environmental mycobacteria

**DOI:** 10.1186/1746-6148-3-23

**Published:** 2007-09-27

**Authors:** Anita L Michel, Lin-Mari de Klerk, Nico C Gey van Pittius, Rob M Warren, Paul D van Helden

**Affiliations:** 1 Tuberculosis Laboratory, ARC-Onderstepoort Veterinary Institute, Private Bag x05, Onderstepoort 0110, South Africa; 2 Game Capture Unit, South African National Parks, Private Bag x402, Skukuza 1350, South Africa; 3 DST/NRF Centre of Excellence in Biomedical Tuberculosis Research, US/MRC Centre for Molecular and Cellular Biology, Department of Biomedical Science, Faculty of Health Sciences – Stellenbosch University, PO Box 19063, Tygerberg 7505, South Africa

## Abstract

**Background:**

African buffaloes are the maintenance host for *Mycobacterium bovis *in the endemically infected Kruger National Park (KNP). The infection is primarily spread between buffaloes via the respiratory route, but it is not known whether shedding of *M. bovis *in nasal and oral excretions may lead to contamination of ground and surface water and facilitate the transmission to other animal species. A study to investigate the possibility of water contamination with *M. bovis *was conducted in association with a BCG vaccination trial in African buffalo. Groups of vaccinated and nonvaccinated buffaloes were kept together with known infected in-contact buffalo cows to allow natural *M. bovis *transmission under semi-free ranging conditions. In the absence of horizontal transmission vaccinated and control buffaloes were experimentally challenged with *M. bovis*. Hence, all study buffaloes in the vaccination trial could be considered potential shedders and provided a suitable setting for investigating questions relating to the tenacity of *M. bovis *shed in water.

**Results:**

Serial water samples were collected from the drinking troughs of the buffaloes once per season over an eleven-month period and cultured for presence of mycobacteria. All water samples were found to be negative for *M. bovis*, but 16 non-tuberculous *Mycobacterium spp. *isolates were cultured. The non-tuberculous *Mycobacterium *species were further characterised using 5'-16S rDNA PCR-sequencing, resulting in the identification of *M. terrae*, *M. vaccae *(or *vanbaalenii*), *M. engbaekii*, *M. thermoresistibile *as well as at least two species which have not yet been classified.

**Conclusion:**

The absence of detectable levels of *Mycobacterium bovis *in the trough water suggests that diseased buffalo do not commonly shed the organism in high quantities in nasal and oral discharges. Surface water may therefore not be likely to play an important role in the transmission of bovine tuberculosis from buffalo living in free-ranging ecosystems. The study buffalo were, however, frequently exposed to different species of non-tuberculous, environmental mycobacteria, with an unknown effect on the buffaloes' immune response to mycobacteria.

## Background

Tuberculosis in wildlife, caused by *Mycobacterium bovis*, has emerged as an increasingly important disease of free-ranging wildlife populations [1-3]. The African buffalo (*Syncerus caffer*) has established itself as a maintenance host for *M. bovis *in two of South Africa's largest conservation areas, namely the Kruger National Park (KNP) and the Hluhluwe iMfolozi Park (HiP) [4,5]. Transmission of *M. bovis *between herd members occurs most frequently by aerosol, whereas spillover to other species requires different modes of transmission [6]. Predators and scavengers alike contract the infection commonly by ingestion of infected tissues [3]. Other pathways may apply only to particular animal species. The secretion of infectious pus from draining fistulae of parotid lymph glands, for example, has been suggested as a mode of transmission between greater kudu (*Tragelaphus strepsiceros*) [7], as well as between cattle and kudu [8]. Contaminated faeces have been implicated in the spread of bovine tuberculosis (BTB) within a troop of baboons (*Papio ursinus*) [9]. Hence, environmental *M. bovis *contamination may be a side effect of events leading to spillover or it may be the cause of spillover itself. If pathogenic microorganisms can retain their viability for some time outside the animal host, environmental sources could play a significant role in their spread to a wide range of animal species from different habitats and ecological niches. To this effect, it has been shown that *M. bovis *can survive for 42 days in tissues with lesions and up to four weeks in faecal material from buffalo [10]. The tenacity of tubercle bacilli in effluents from sanatoria and dairies and its significance in the spread of infection to cattle were major public health concerns prior to eradication of BTB in Europe [11,12].

No information is, however, available on the role of surface water in the epidemiology of bovine tuberculosis in an endemically infected ecosystem, especially where limited water sources cause a variety of animal species to gather in high densities for most of the year.

The present study was conducted during a BCG vaccination trial in buffalo involving *M. bovis *challenge. We used this opportunity to determine whether naturally and/or experimentally infected buffalo were shedding detectable levels of *M. bovis *into the drinking water, and if so, to provide an estimate of the organism's tenacity.

## Results

### Animals

The results of macroscopic and culture examination of all surviving study animals are summarised in Table 1. *M. bovis *infection was confirmed by culture and subsequent PCR identification of acid-fast isolates in 14 of the 24 surviving in-contact buffalo cows. Three culture positive buffalo did not yield macroscopic lesions and two buffalo with lesions in a single lymph node were culture negative. A total of 13 buffaloes presented with macroscopic lesions, two of which had lesions restricted to lymph nodes. Eleven cows presented with macroscopic lung lesions varying from pinpoint foci in two cases, to disseminated tuberculous pneumonia in nine animals (de Klerk, unpublished data).

**Table 1:** Bovine tuberculosis culture and lesion status of the different groups of study buffalo

No. of buffalo	Group	No. of buffalo surviving	Culture positive	No. buffalo with lesions
27	Experimental	27	17	15
27	In-contact	24	14	13
11	Calves*	10	2	2
Total	*	61	33	30

*One calf which died before the end of the study presented with lesions consistent with pneumonia and yielded *M. bovis *on culture of lung tissue

From the experimental group, fourteen buffaloes showed visible lesions in mainly the lymph nodes of the head, with only three having secondary spread to thoracic lymph nodes. One additional buffalo had a single lung lesion. However, 17 buffaloes yielded *M. bovis *on culture. *M. bovis *infection was confirmed in two out of the ten surviving calves, both showed involvement of the thoracic lymph nodes. Another calf, which died a few weeks after birth, yielded *M. bovis *from lung tissue.

### Isolation and identification of *Mycobacterium spp. *by polymerase chain reaction (PCR)

In contrast to the tissue samples, *M. bovis *was not cultured from any of the water samples except the spiked water used for quality control. Four inoculated trough water samples containing *M. bovis *concentrations ranging from 2·10^5 ^to 2·10^2^/ml yielded abundant growth of *M. bovis *after three weeks for the highest and after eight weeks for the lowest concentration.

**Figure 1:**
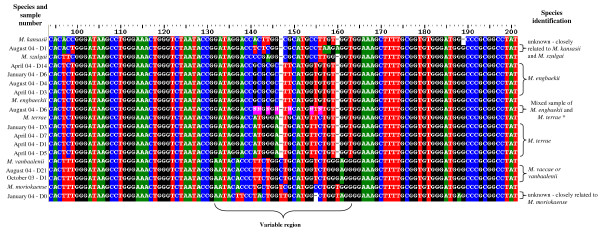
**Multiple sequence alignment of a part of the 16S rRNA gene of Mycobacterium species isolated from water samples**. Sample sequences obtained through PCR sequencing of the 16S rRNA gene were aligned with those of type strains of species resembling the isolated mycobacteria. Species-specific variable region is indicated. * – mixed sample in August identified by two different overlapping chromatograms at the positions indicated by N and highlighted in pink. Chromatogram 1 – ATGGGATGCATGTTC = *M. terrae *Chromatogram 2 – GCGCGCTTCATGGTG = *M. engbaekii*

However, 16 other mycobacterial isolates were recovered from water samples (Table 2) and two others were cultured from lymph nodes of vaccinated buffalo. These 18 *Mycobacterium spp. *isolates were acid-fast on microscopic smear examination but failed to amplify the expected 372 bp product in the PCR protocol used to identify *M. tuberculosis *complex bacteria (data not shown). Subsequent analysis using 5'-16S rDNA PCR-sequencing revealed that these *Mycobacterium spp. *belonged to the species *M. terrae*, *M. engbaekii*, *M. vaccae *(or *vanbaalenii*) and two previously unidentified species closely related to *M. moriokaense *and *M. kansasii *(and *M. szulgai*), respectively (Table 2 and Figure 1). The mycobacterial species isolated from the buffalo tissues were identified as *M. thermoresistibile *and an unidentified species closely related to *M. moriokaense *(Fig. 1). Three NTM isolates could not be further identified to species level.

**Table 2:** Culture of water samples collected from drinking troughs of buffalo

Sampling occasion		Culture result	Water temp in exptl trough. (°C)
1	October 2003	N	25
	Day 1	*M. vaccae or M. vanbaalenii*	35
	Day 2	N	37
	Day 3	N	33
	Day 4	NTM*	36.5
	Day 5	N	33
	Day 6	N	25
	Day 7	N	31
	Day 14	N	46
	Day 21	N	42.5

2	January 2004	Unknown *Mycobacterium *species closely related to *M. moriokaense*	28
	Day 1	P/C	37
	Day 2	C	36.5
	Day 3	*M. terrae*	30
	Day 4	N	29
	Day 5	P/C	34
	Day 6	*M. engbaekii*	36
	Day 7	N	34
	Day 14	P/C	30
	Day 21	P/C	32

3	26 April 2004	C	23.6
	Day 1	*M. terrae*	20.0
	Day 2	N	22.0
	Day 3	*M. engbaekii*	26.5
	Day 4	N	26.5
	Day 5	*M. terrae*	26.0
	Day 6	NTM*	26.5
	Day 7	*M. terrae*	24.5
	Day 14	*M. engbaekii*	23.0
	Day 21	N	23.6

4	02 August 04	N	18.5
	Day 1	Unknown *Mycobacterium *species closely related to *M. kansasii *and *M. szulgai M. terrae*	24
	Day 2	N	25
	Day 3	No sample	N/A
	Day 4	M. engbaekii	24
	Day 5	N	25
	Day 6	Mixed culture of *M. engbaekii *and *M. terrae*	26
	Day 7	NTM*	25
	Day 14	N	26.5
	Day 21	*M. vaccae or M. vanbaalenii*	19.5

* Non-tuberculous mycobacterial species not further identified

P/C: cultures partially contaminated

C: cultures contaminated

N: negative culture result

## Discussion

To assess the risk of *M. bovis *spillover from buffalo to other species it is critical to determine the mode, frequency and level of shedding by infected buffalo herds. Neill et al. [14,15], concluded from experimental infections in calves that excretion of *M. bovis *in nasal mucus is a consistent feature in all infected cattle and can continue for weeks and even months. Furthermore, shedding rates ranging from 6% to 20% were found among naturally infected, tuberculous cattle in different countries, whereby the occurrence of lung lesions may be very low [14]. If the same was true for African buffalo, for which lesion types and distribution are generally comparable to those in cattle, they could possibly spread bovine tuberculosis by contaminating surface water such as water holes, dams and pools formed in stagnant rivers, as they commonly spend extended periods in and along the various water points. Especially large buffalo herds with high *M. bovis *infection rates as documented for the southern region of the Kruger National Park [3], could pose a significant risk to all susceptible species in the area.

Based on the abovementioned shedding rate for cattle, our study could have contained at least two to six shedders among the 31 tuberculous buffalo at any time during the study (Table 1). The actual conditions for shedding in buffalo were, however, more favorable than reported for cattle [14], since our study population included nine in-contact buffalo cows with advanced ('open') lung lesions, which is considered a sign of infectiousness in cattle [16,17]. If *M. bovis *shedding was an intermittent but common feature in infected buffalo, we had expected the *M. bovis *load in the trough water to be well above the confirmed detection limit of bacterial culture.

Our study did, however, not provide any indication of detectable amounts of *M. bovis *being present in the water troughs. Despite a degree of sampling uncertainty e.g. mycobacteria trapped in sediments or biofilms may have escaped sampling, we believe that the culture method used was suitable since it supported isolation of 16 *Mycobacterium spp*. isolates and of *M. bovis *from all spiked water samples. We are therefore of the opinion that shedding of *M. bovis *in nasal and oral discharges is an infrequent event in African buffalo, possibly limited to animals with clinical signs or to very low bacterial loads below the detection limit. This finding suggests a low to negligible risk for buffalo to serve as transmitters of *M. bovis *via water under free-ranging conditions.

Our conclusions are furthermore supported by the fact that no evidence of horizontal transmission of *M. bovis *between in-contact cows and the experimental buffalo could be demonstrated (results not shown). This includes potential spread by water or aerosolised droplets. Spread by aerosolised droplets may be dependent on frequent, close physical contact and social interactions, which, although well described characteristics in buffalo behaviour, were not observed between the two study groups. The in-contact and experimental buffalo had been sourced from different herds. Apart from grazing and resting in relative proximity to each other, the two groups remained separate social entities throughout the vaccination trial. This observation is important as it may indicate that social and behavioural patterns are key determinants in driving transmission within and between buffalo herds and warrants more in-depth investigations.

Unlike members of the *M. tuberculosis *complex, NTMs are rarely associated with invasive disease, but they may temporarily colonise the host and cause transient infection accompanied by non-specific stimulation of the host's immune system [18]. In our study we isolated an unidentified *Mycobacterium *species closely related to *M. moriokaense *from both the trough water and lymph node tissue from one of the buffalos, suggesting the environment as the source of infection.

The specific effect of this particular, or other, NTM species on the immune response of buffalo is unknown at this stage. In both, cattle and buffalo, environmental mycobacteria have been suspected to cause non-specific sensitisation to the tuberculin skin test [19,20]. Corner et al. [21,22] showed that cattle inoculated with atypical mycobacteria isolated from either soil or bovine origin developed a significant level of sensitivity to both bovine and avian PPD in the tuberculin skin test, The immune response lasted for between four and ten weeks and was generally higher for avian PPD than bovine PPD. Demangel et al. [23] have implied a potential adverse effect of environmental mycobacteria on vaccination efficacy of BCG, depending on the extent to which these mycobacteria share cross-reacting antigens with the vaccine. In calves, sensitisation with environmental mycobacteria prior to vaccination had an antagonistic effect on BCG efficacy [24]. Initial vaccination trials using BCG in buffalo did not yield a significant reduction of infection, questioning the efficacy of BCG in these animals (de Klerk unpublished data) and raising the question whether this effect might be due to the presence of NTMs. Our microbiological examination of water, pumped from a tributary of the Sabie River into the water trough for the study buffaloes, yielded five species of NTM, including two previously unidentified species (Table 2). *M. vaccae *and *M. terrae *are reportedly among the most frequently isolated organisms from fresh water [25], along with *M. engbaekii *and a number of unclassified mycobacteria [26]. Their natural habitat, however, is more likely to be wet soil [27], which may suggest that the NTMs, especially *M. terrae*, did not all originate from the river water but could have been introduced via the soiled muzzles or feet of the buffalo while drinking. Favourable water temperatures throughout the experiments (Table 2) and the presence of sufficient nutrients in river water are known to facilitate replication of mycobacteria [27].

## Conclusion

The findings of this study suggest that contamination of surface water by infected buffalo may not be likely to play a significant role in the spread of *M. bovis *in a free-ranging ecosystem. The study also demonstrated that buffalo were exposed to different environmental NTMs in river-water without producing any signs of infection or disease.

Further studies will be required to investigate the potential effects of these and other NTM species on the immune response of buffalo especially in the context of BTB control strategies involving vaccination and diagnostics.

## Methods

### Study site

The present study was dovetailed with a BCG vaccination trial in buffalo, and was carried out in a 100 hectare fenced camp with natural habitat near Skukuza in the KNP. Two concrete drinking troughs (inner troughs) with a capacity of 500 liters each, were located in an enclosure situated within one corner of the camp and were the only permanent water source for the buffalo during the trial. Fresh water was pumped daily from a tributary of the nearby Sabie River to replenish the water in both drinking troughs. A separate concrete trough (experimental trough) with a capacity of approximately 250 liters was located next to the inner troughs but on the outside of the enclosure and camp. This trough was used for collection of serial water samples as described below. Access to the inner water troughs was restricted for several hours before each new sampling experiment to ensure that all buffaloes would consume water before sampling took place later on that same day.

### Animals

A trial to evaluate the efficacy of BCG vaccination in African buffalo was conducted between January 2003 and November 2004 with prior approval by the Animal Care and Use Committee of the South African National Parks. Before the start of the project twenty-seven PPD skin test and interferon gamma negative buffalo (experimental buffalo), aged about two years, were translocated from the northern part of KNP into a holding facility (boma) at Skukuza. Prior to introduction into a 100 hectare camp, 14 animals were randomly selected and vaccinated twice, six weeks apart, with BCG (Pasteur strain P1172) via the intramuscular route (de Klerk, unpublished data). The initial vaccination protocol anticipated natural *M. bovis *challenge from close contact with infected herd members. For this purpose a group of 27 adult buffalo cows (in-contact buffalo) was captured from a high prevalence herd in the south of the KNP and introduced into the same camp to join up with the 27 experimental buffalo. After a period of eleven months without any evidence (skin test, interferon gamma test) of horizontal transmission of *M. bovis *to the nonvaccinated buffalo, both the vaccinated and nonvaccinated groups were challenged with a field strain of *M. bovis *via the intra-tonsilar route in January 2004 [13]. For the purpose of the present study the vaccination status of the experimental buffalo was considered insignificant and hence no distinction is made hereafter between the two treatment groups. Twenty-four of the 27 in-contact buffalo cows as well as all 27 experimental buffalo survived. Three cows died of undetermined causes. During the trial period 16 calves were born to the in-contact cows, of which ten survived. Three months after the last water sampling was conducted, all buffalo (n = 61) were slaughtered in November 2004.

### Water sample collection plan

One sampling experiment was conducted each in October 2003, as well as in the months of January, April, and August 2004 (Table 2). The experimental troughs were emptied and dried between sampling experiments. At the start of each sampling experiment, the buffalo were allowed to drink from both inner troughs and subsequently moved out of the enclosure. Following mixing of the water and collection of the first water sample (Table 2), approximately 100 liters of water was transferred manually from each inner trough into the experimental trough outside the fence, using a bucket. The water temperature in both troughs was recorded daily. Each experiment was designed to determine the survival time of potentially excreted *M. bovis*, by collecting ten serial water samples into 400 ml sterile containers. On day 1 the water sample was taken from the inner trough within two hours after buffalo contact. Thereafter water samples were collected from the experimental trough on a daily basis up to day 7 as well as on day 14 and day 21. The water samples were stored at -minus 20°C until transferred to the Tuberculosis laboratory at the ARC-Onderstepoort Veterinary Institute (OVI) for culture. For quality control purposes, four aliquots of 50 ml trough water each, were spiked with serial dilutions from 10^7 ^to 10^4 ^organisms from a *M. bovis *field strain and frozen until processing.

### Tissue sample collection plan

At slaughter, a standard set consisting of nine lymph node samples was collected for histopathology and culture from each buffalo. The samples included lymph nodes of the head (incl. tonsils), thorax, abdomen and carcass. Samples were also collected from any other tissues with visible lesions. All tissue samples for culture were individually packed in sterile containers and frozen at minus 20°C until processed in the Tuberculosis laboratory at the OVI.

### Bacterial isolation

Tissue samples were processed and cultured according to the protocol described by Bengis et al. [6]. A modification of the standard protocol was used to isolate *M. bovis *from the water samples and the quality control samples. Briefly, the water was centrifuged at 3500 rpm for 10 min and the pellet resuspended in 25 ml of sterile, double distilled water. Decontamination was effected by adding 25 ml of sodium hydroxide (4% w/v). The mixture was left for 10 minutes before centrifugation for 15 min at 3500 rpm. The pellet was neutralized by adding 5% oxalic acid for 15 minutes, followed by centrifugation as before. The pellet was mixed and inoculated onto four slants of Lőwenstein-Jensen medium, two of which contained pyruvate to facilitate growth of *M. bovis*. All cultures were evaluated for colony growth on a weekly basis up to 10 weeks. Cultures slants, which showed contamination on less than 50% of the medium surface, were classified as partially contaminated. These cultures were maintained unless the contamination covered more than 50% of the medium slant in which case it was discarded for contamination.

### Identification of *Mycobacterium spp. *by polymerase chain reaction (PCR)

All acid-fast isolates were subjected to a PCR assay specific for *M. tuberculosis *complex bacteria [28]. All isolates, which failed to yield the expected 372 bp amplification product, were subjected to a 5'-16S rDNA PCR-sequencing assay, which is able to identify non-tuberculous *Mycobacterium spp*. [29].

## Competing interests

The author(s) declare that they have no competing interests.

## Authors' contributions

ALM designed and supervised the experiment as well as the bacteriological analyses and drafted the manuscript. LMDK was the project leader of the BCG trial and performed the field work. NGVP performed the sequence analysis. NGVP and RW added value through introduction of critical technical considerations. PVH was instrumental in the inter-institutional collaboration and contributed overall to the manuscript. All authors read and approved the final manuscript.
